# Supplementary material for: Improvement of Fertilization Capacity and Developmental Ability of Vitrified Bovine Oocytes by JUNO mRNA Microinjection and Cholesterol-Loaded Methyl-β-Cyclodextrin Treatment
Source: Int J Mol Sci. 2022 Dec 29;24(1):590. doi: 10.3390/ijms24010590 (PMC9820539; doi:10.3390/ijms24010590)
Supplement: Supplementary file 1 [file ijms-24-00590-s001.zip › ijms-2049362 supplementary figure captions.pdf]

Figure S1. The mass spectrograms of disulfide bond of JUNO protein in fresh bovine oocytes. A: The mass spectrogram of disulfide bond at location 27-125 of JUNO protein in fresh bovine oocytes. B: The mass spectrogram of disulfide bond at location 18-165 of JUNO protein in fresh bovine oocytes. C: The mass spectrogram of disulfide bond at location 18-179 of JUNO protein in fresh bovine oocytes. D: The mass spectrogram of disulfide bond at location 99-165 of JUNO protein in fresh bovine oocytes. E: The mass spectrogram of disulfide bond at location 95-165 of JUNO protein in fresh bovine oocytes. F: The mass spectrogram of disulfide bond at location 79-47 of JUNO protein in fresh bovine oocytes. G: The mass spectrogram of disulfide bond at location 79-79 of JUNO protein in fresh bovine oocytes. H: The mass spectrogram of disulfide bond at location 79-99 of JUNO protein in fresh bovine oocytes.

Figure S2. The mass spectrograms of disulfide bond of JUNO protein in vitrified bovine oocytes. A: The mass spectrogram of disulfide bond at location 79-229 of JUNO protein in vitrified bovine oocytes. B: The mass spectrogram of disulfide bond at location 95-95 of JUNO protein in vitrified bovine oocytes. C: The mass spectrogram of disulfide bond at location 79-79 of JUNO protein in vitrified bovine oocytes. D: The mass spectrogram of disulfide bond at location 79-179 of JUNO protein in vitrified bovine oocytes. E: The mass spectrogram of disulfide bond at location 125-142 of JUNO protein in vitrified bovine oocytes.

Figure S3. The mass spectrogram of phosphorylation at location 75 of JUNO protein in vitrified bovine oocytes.

Figure S4. The mass spectrogram of glycosylation at location 174 of JUNO protein in fresh bovine oocytes.

Figure S5. The mass spectrograms of glycosylation of JUNO protein in vitrified bovine oocytes. A: The mass spectrogram of glycosylation at location 225 of JUNO protein in vitrified bovine oocytes. B: The mass spectrogram of glycosylation at location 184 of JUNO protein in vitrified bovine oocytes. C: The mass spectrogram of glycosylation at location 174 of JUNO protein in vitrified bovine oocytes.
