# Supplementary figures and images for: Improvement of Fertilization Capacity and Developmental Ability of Vitrified Bovine Oocytes by JUNO mRNA Microinjection and Cholesterol-Loaded Methyl-β-Cyclodextrin Treatment
Source: Int J Mol Sci. 2022 Dec 29;24(1):590. doi: 10.3390/ijms24010590 (PMC9820539; doi:10.3390/ijms24010590)

A

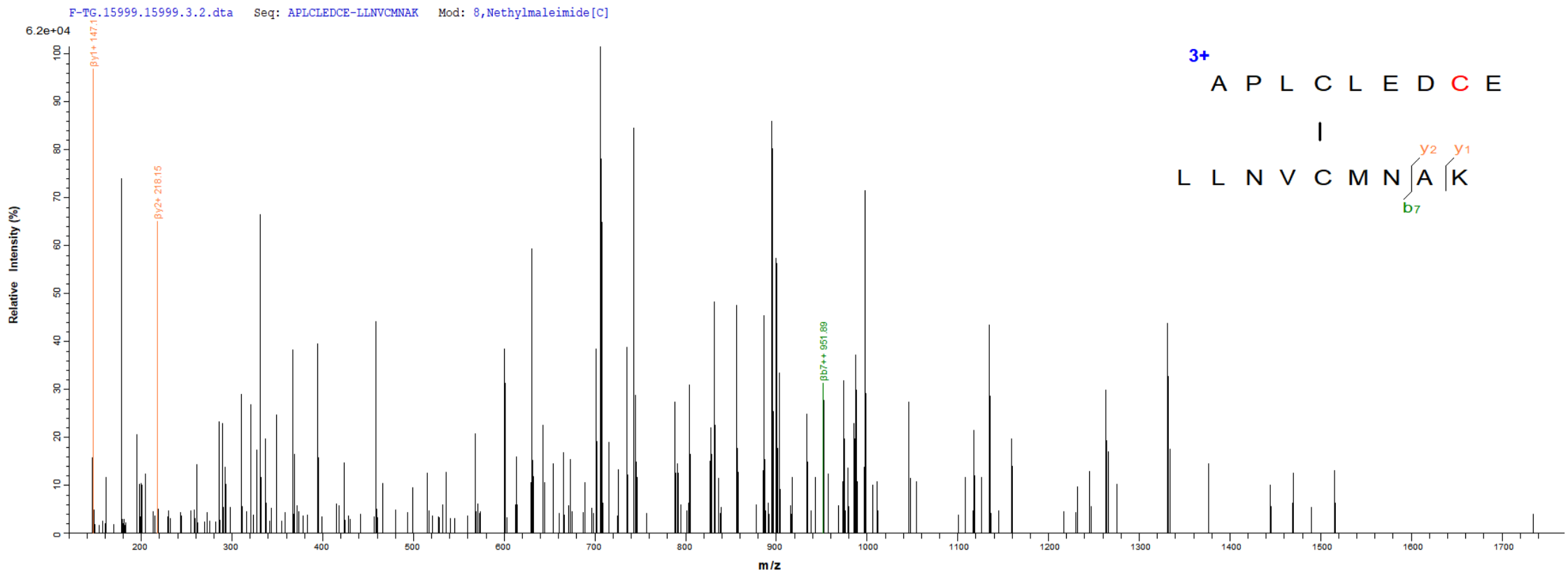

B

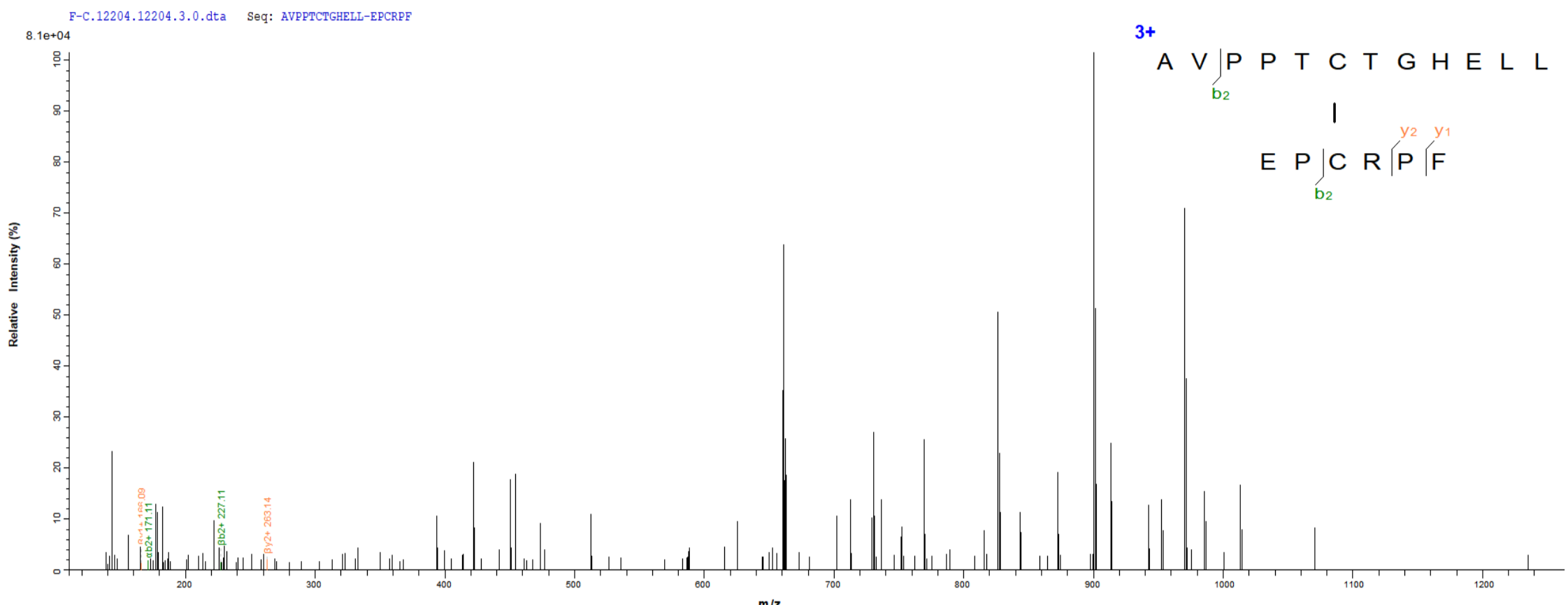

C

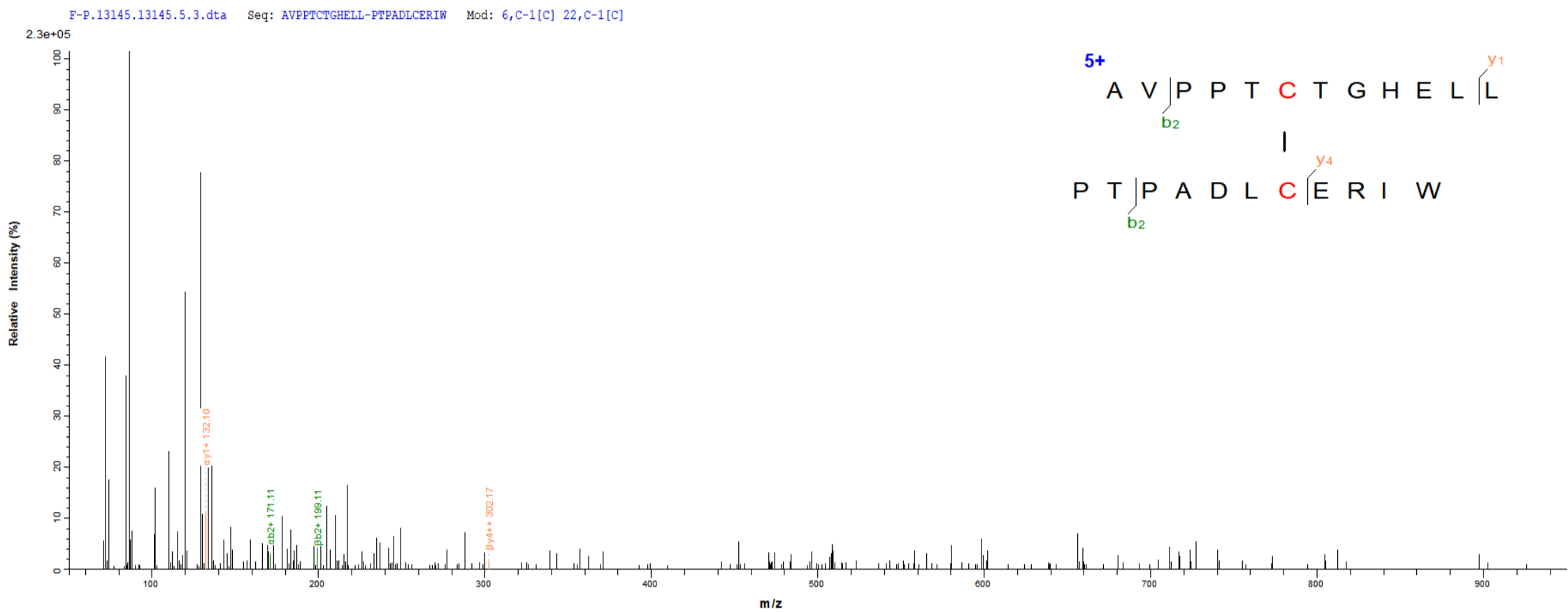

D

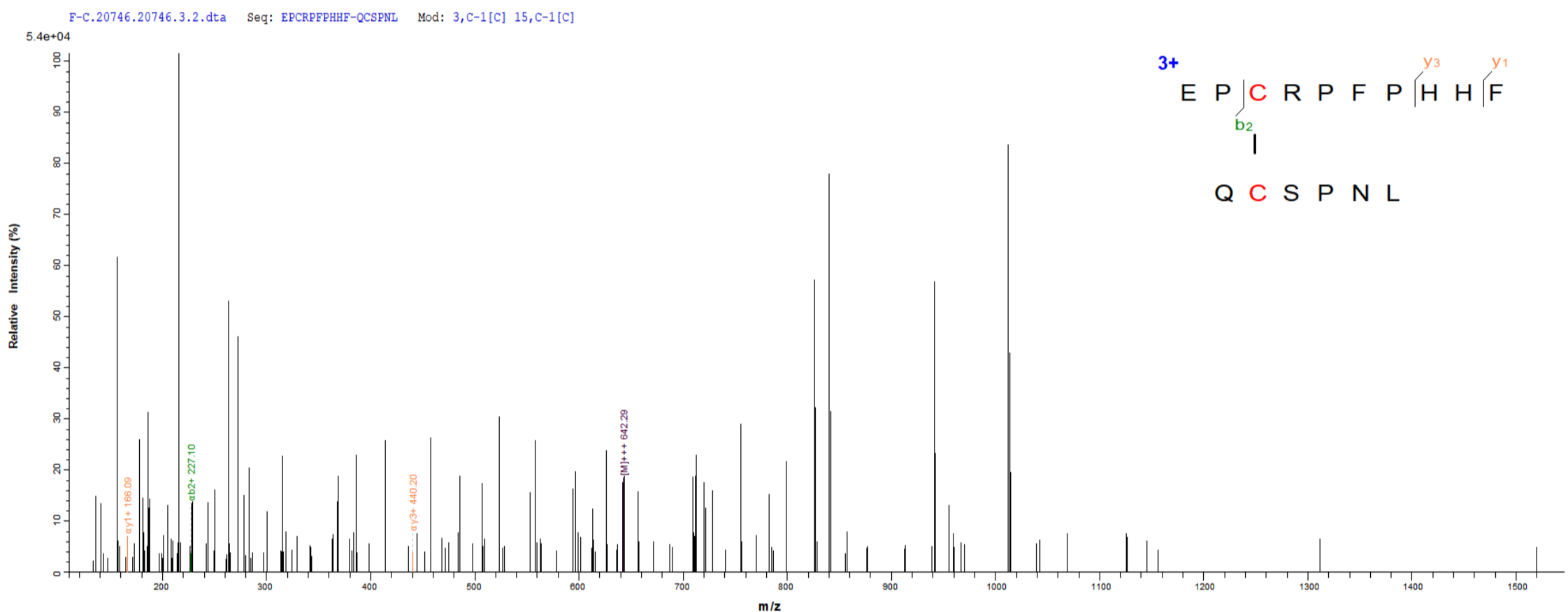

E

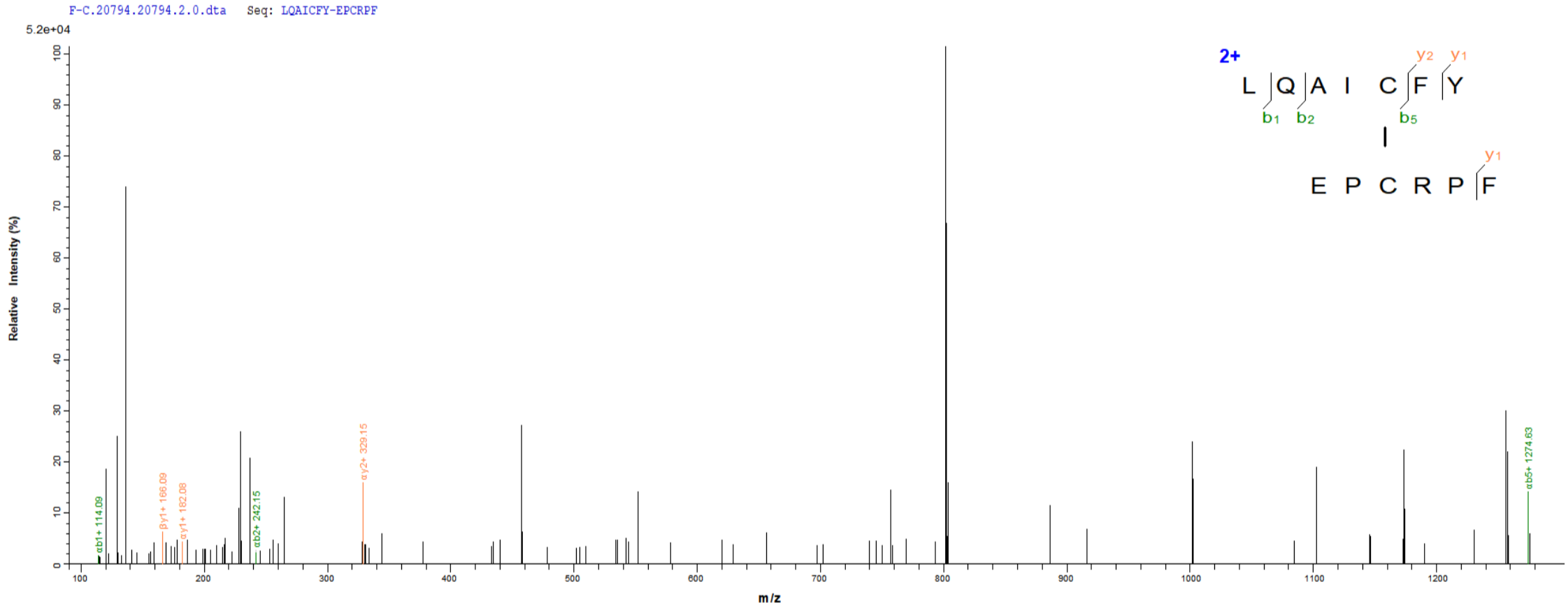

F

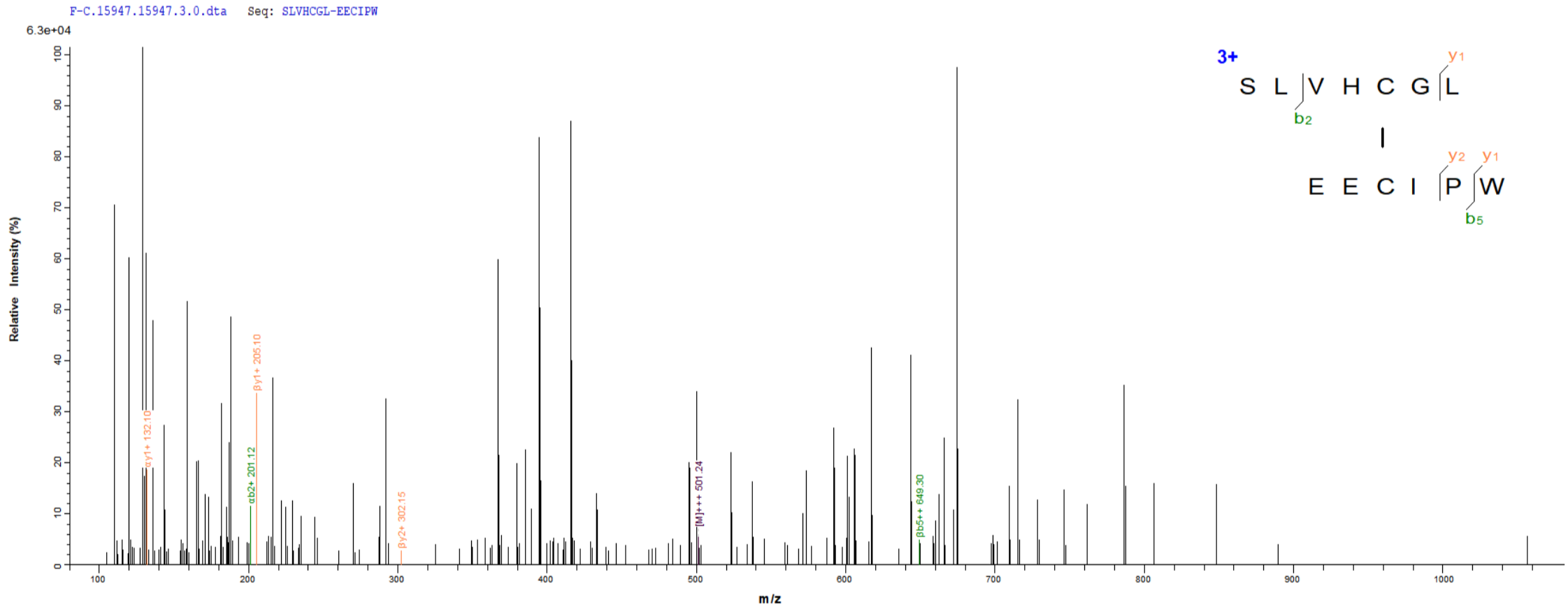

G

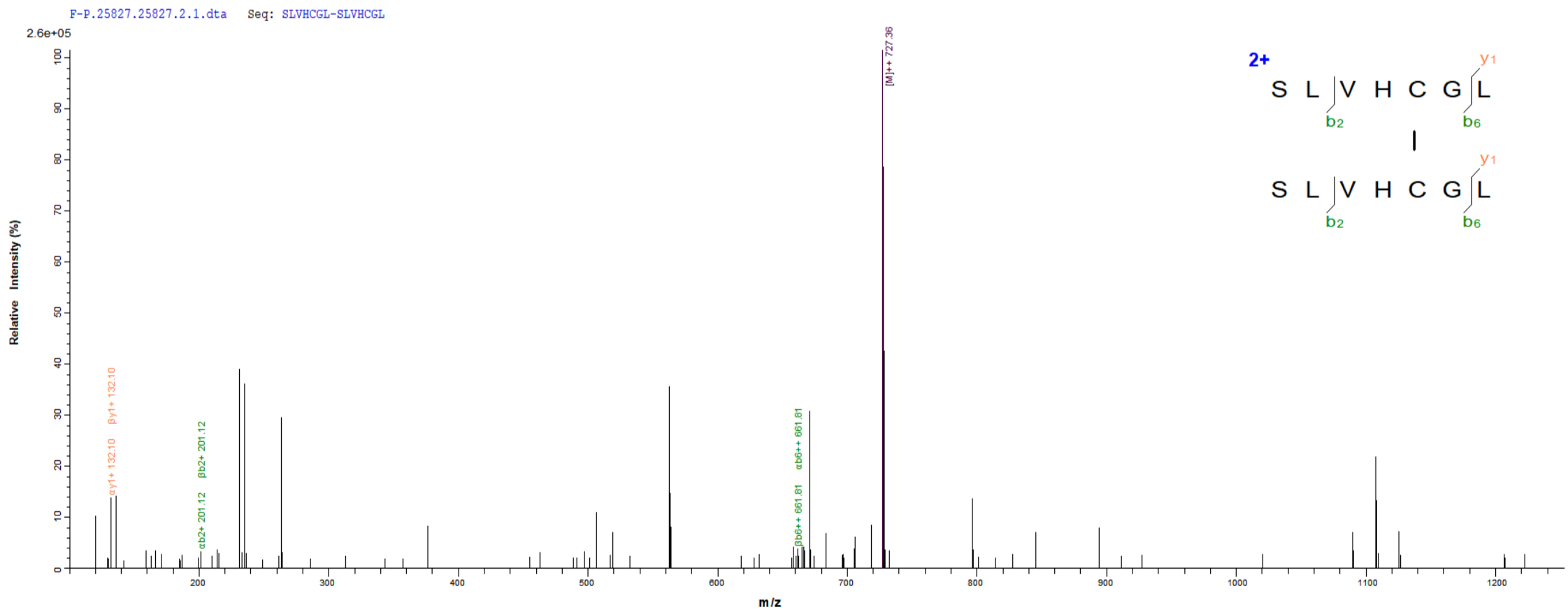

H

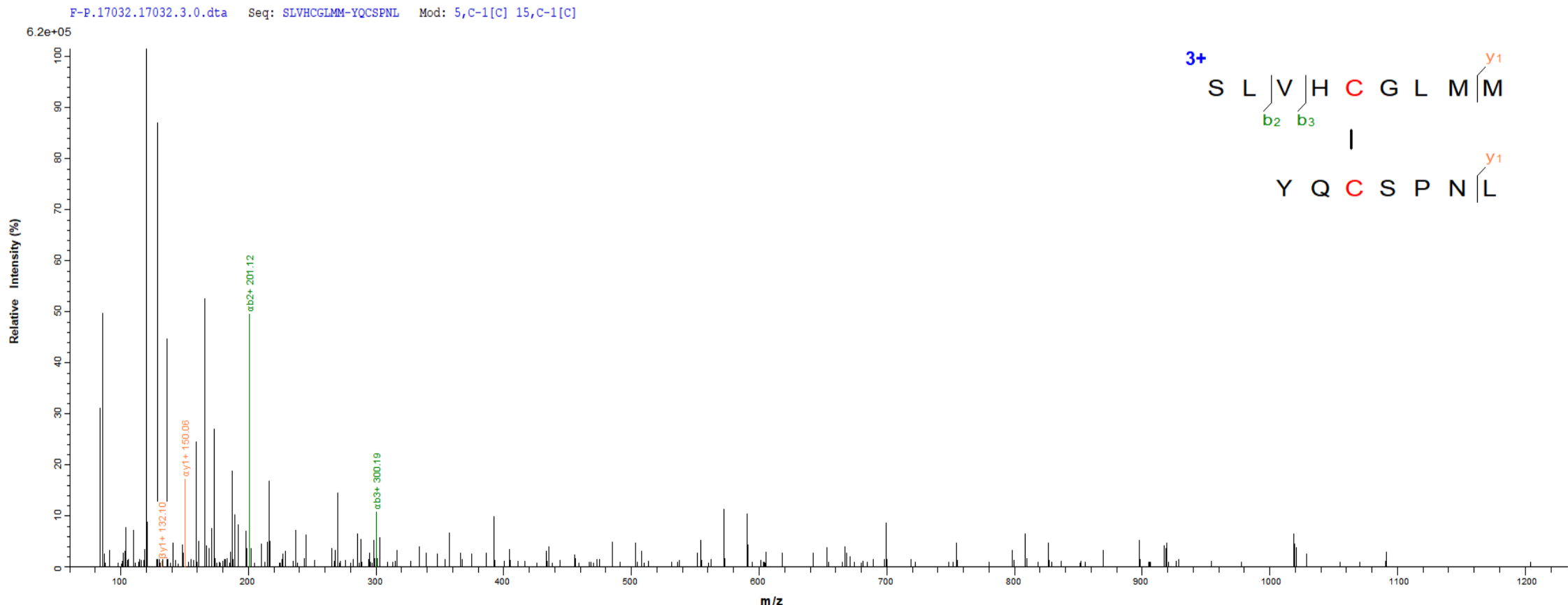

Supplement: Supplementary file 1 [file ijms-24-00590-s001.zip › Fig.S1.pdf]

A

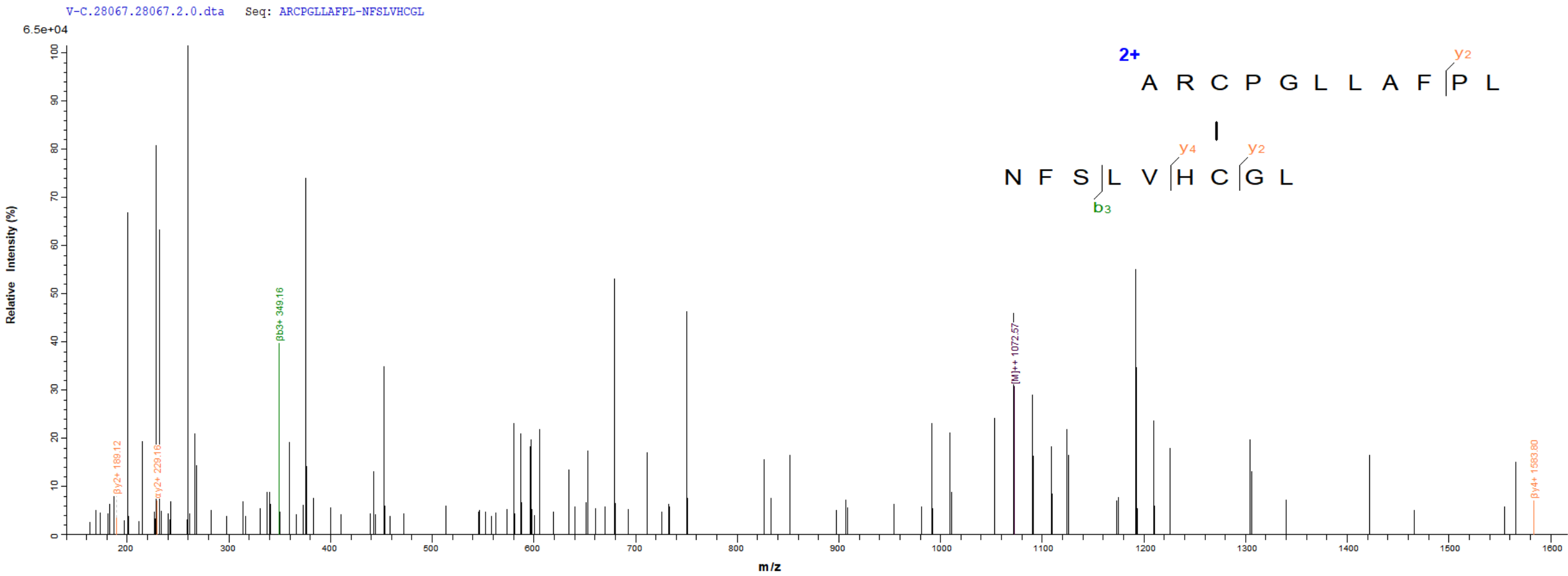

B

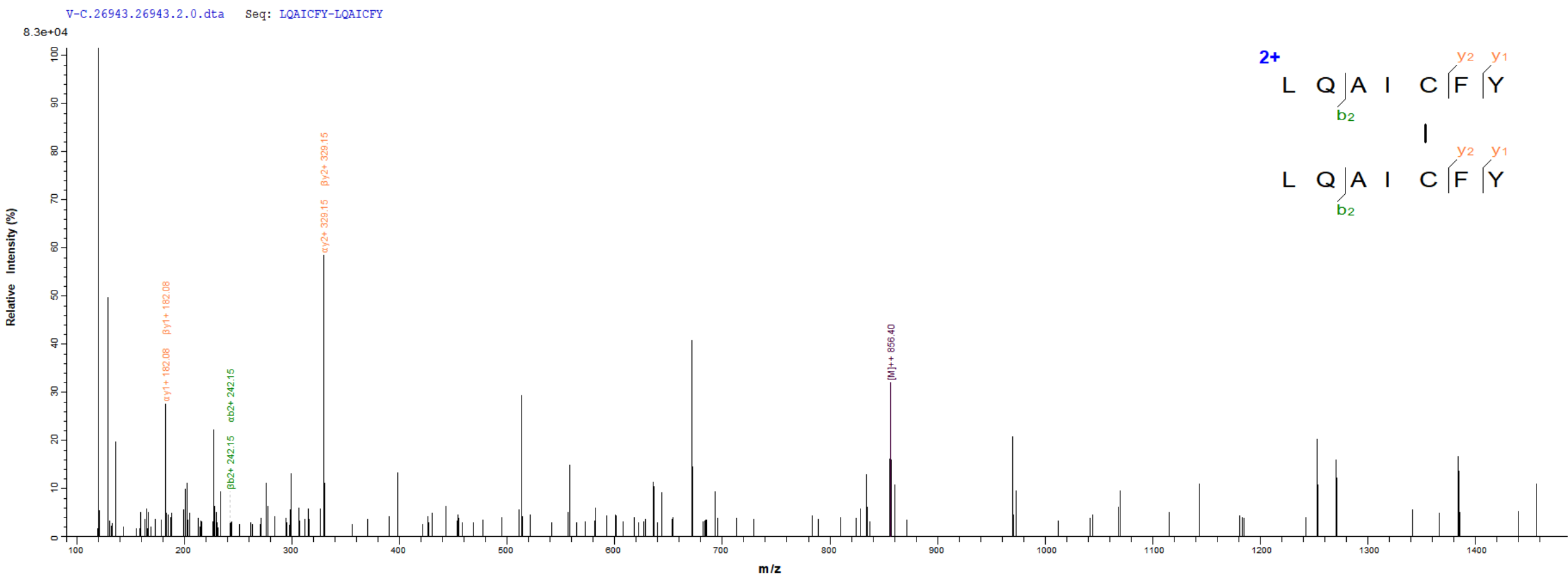

C

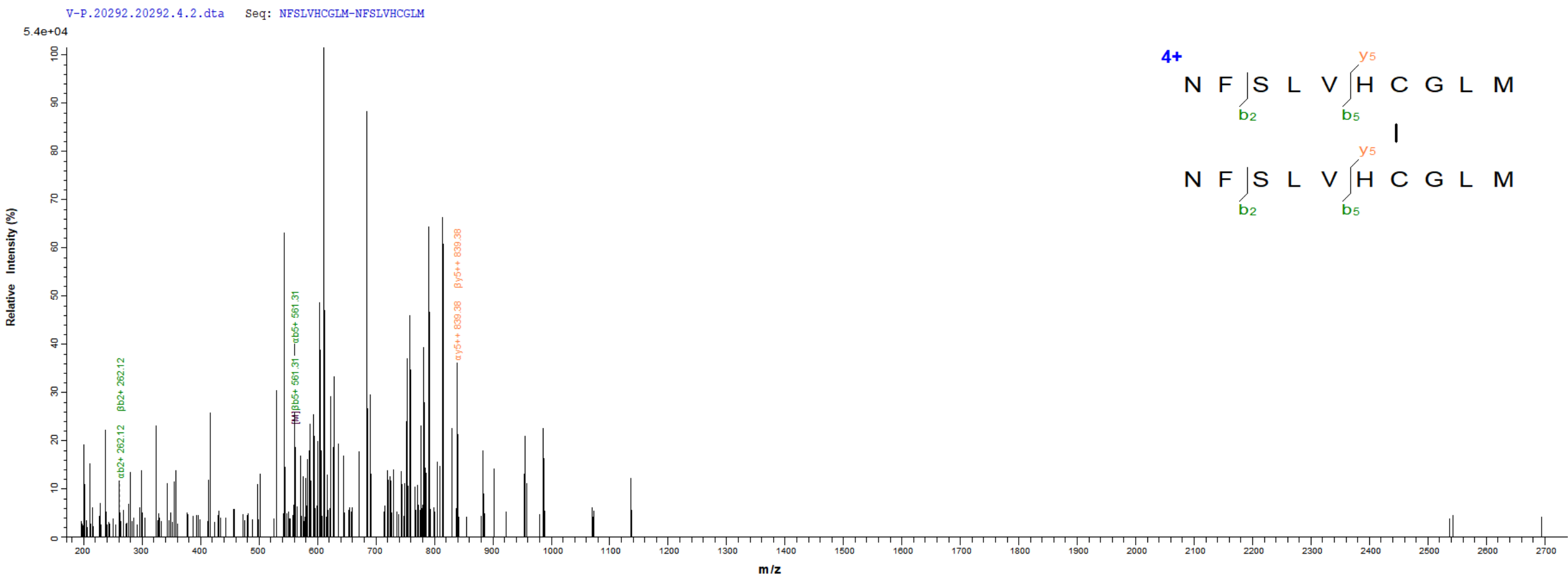

D

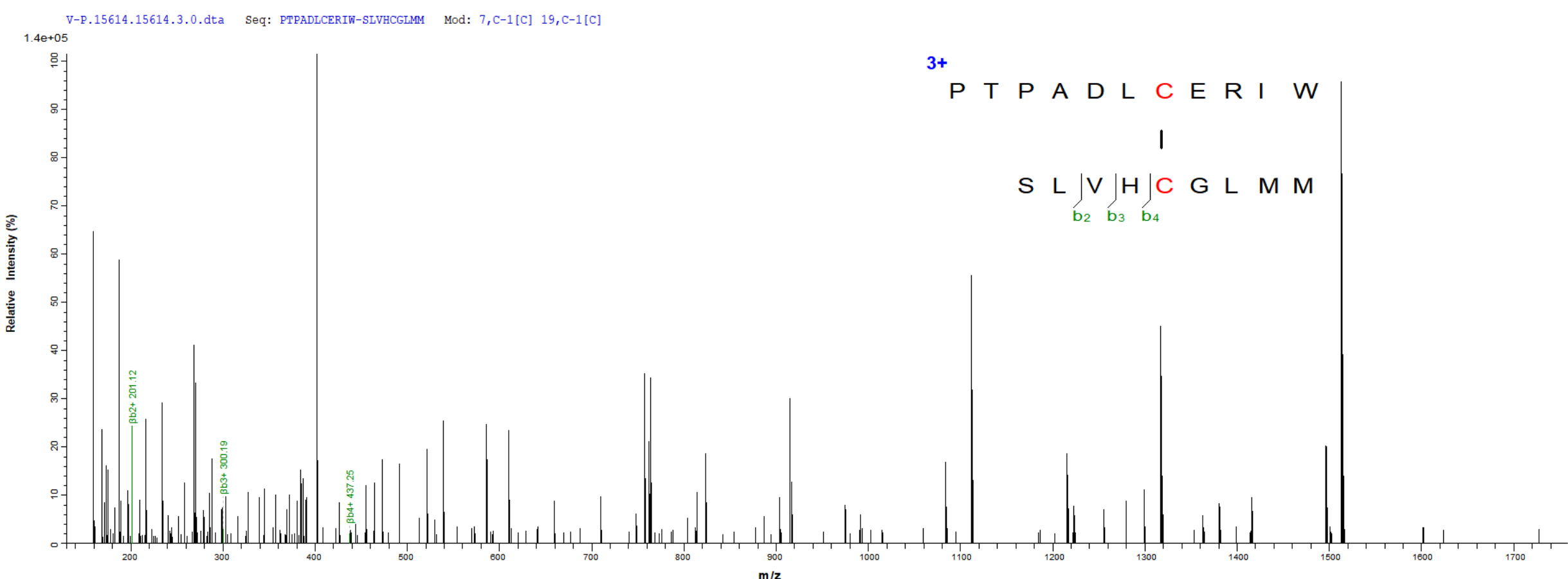

E

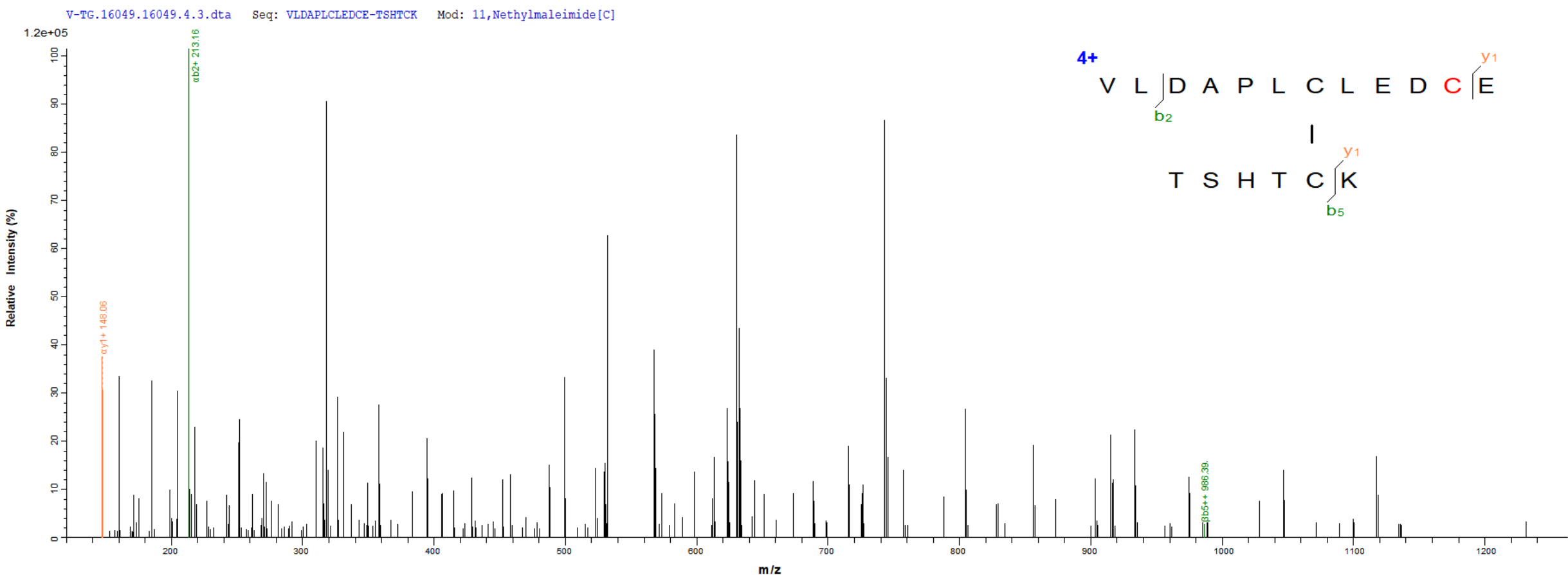

Supplement: Supplementary file 1 [file ijms-24-00590-s001.zip › Fig.S2.pdf]

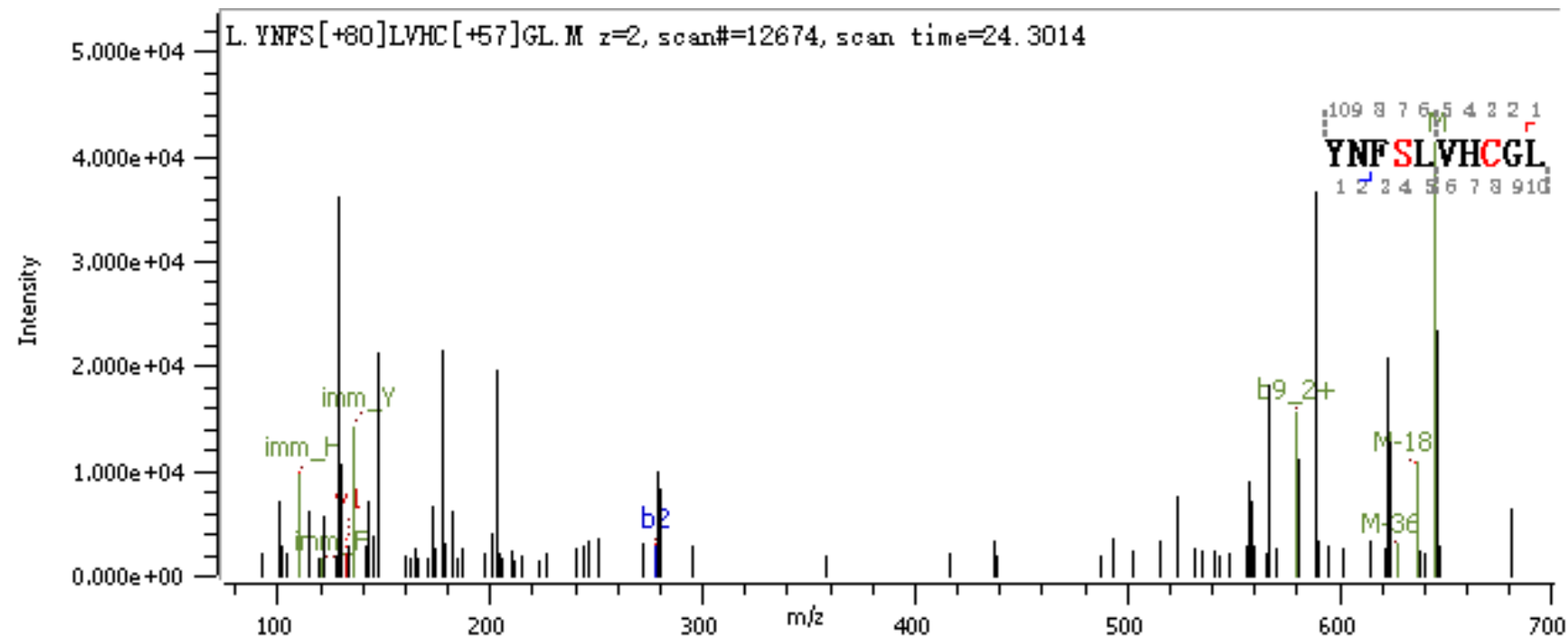

Supplement: Supplementary file 1 [file ijms-24-00590-s001.zip › Fig.S3.pdf]

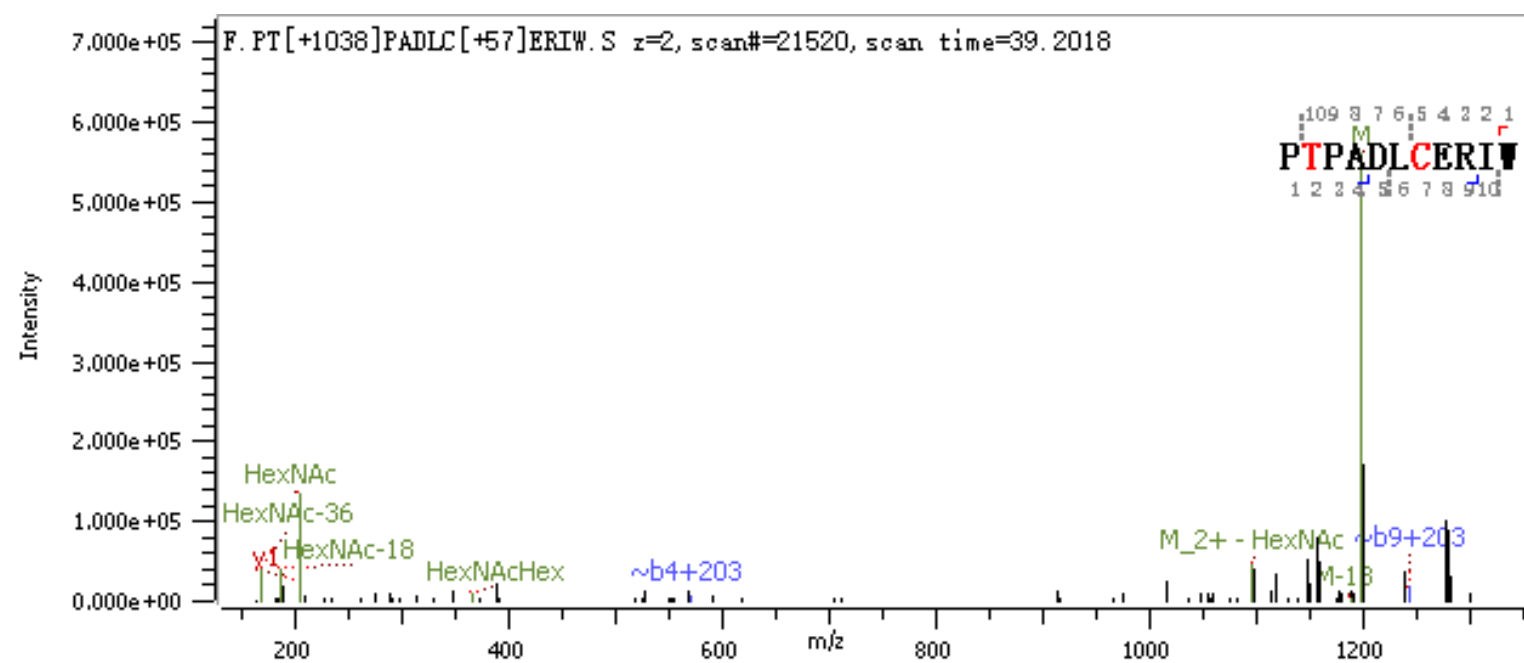

Supplement: Supplementary file 1 [file ijms-24-00590-s001.zip › Fig.S4.pdf]

**A**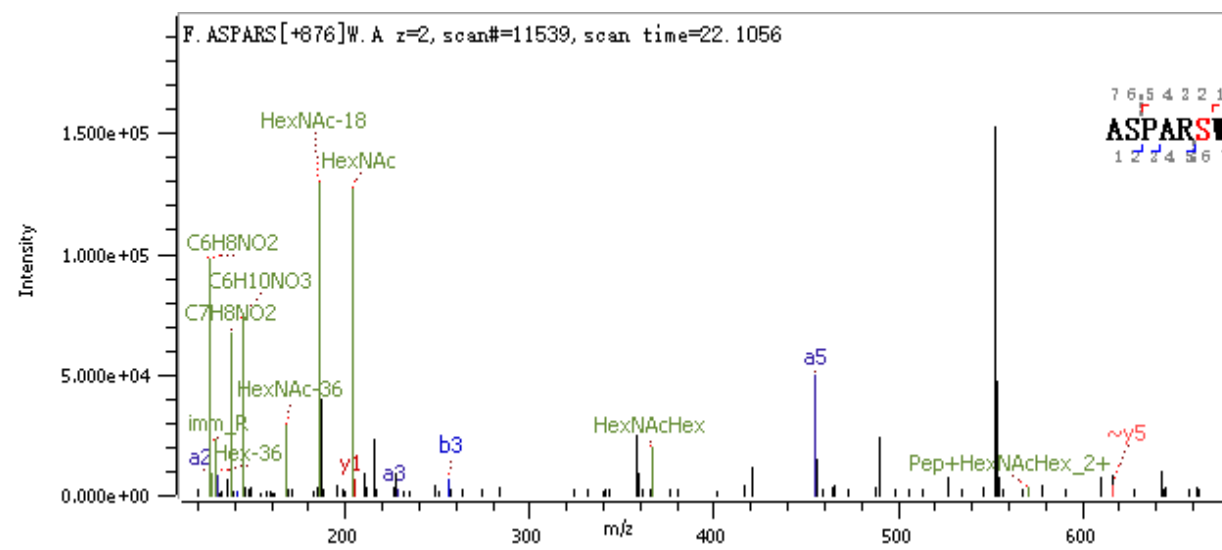**B**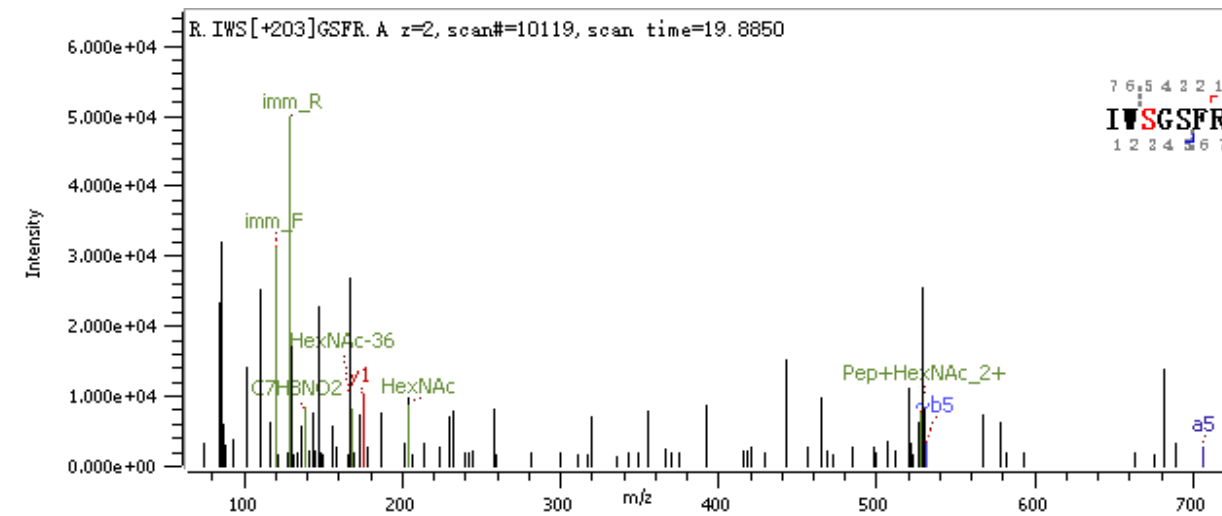**C**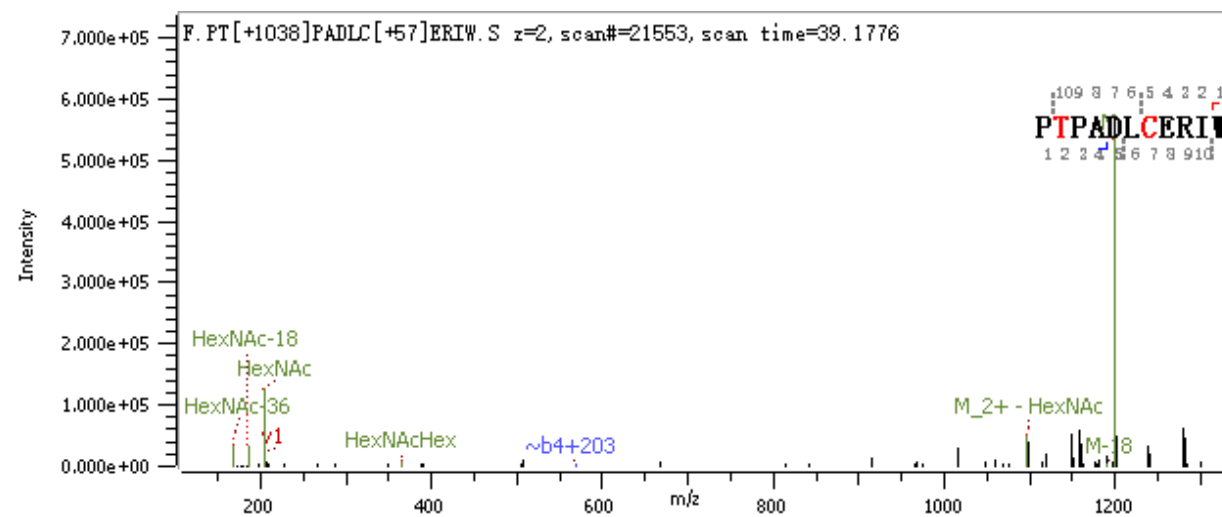

Supplement: Supplementary file 1 [file ijms-24-00590-s001.zip › Fig.S5.pdf]
